# Editorial: Host-pathogen interactions in intracellular bacteria: mechanisms, evasion strategies, and therapeutic insights

**DOI:** 10.3389/fmolb.2026.1919948

**Published:** 2026-07-13

**Authors:** Mehak Zahoor Khan, Akansha Singh, Jasim Khan, Danish Umar, Owais Hakiem

**Affiliations:** 1 Ragon Institute of Mass General Brigham, MIT and Harvard, Cambridge, MA, United States; 2 Rensselaer Polytechnic Institute, Troy, NY, United States; 3 UAB Heersink School of Medicine, University of Alabama at Birmingham, Birmingham, AL, United States; 4 NOMIS Center for Immunobiology and Microbial Pathogenesis, Salk Institute for Biological Studies, La Jolla, CA, United States; 5 Department of Microbiology and Molecular Genetics, University of California Irvine, Irvine, CA, United States

**Keywords:** chlamydia, host pathogen interaction, immune evasion, intracellular bacteria, persistence, transcriptomics, vaccine

Intracellular bacteria exploit host cells as protective niches that facilitate survival, replication, dissemination, and immune evasion, unlike extracellular bacteria, which remain directly exposed to soluble immune mediators and antimicrobial agents. To establish an intracellular niche, they tightly regulate bacterial gene expression and coordinate early events such as adhesion, invasion, and inclusion formation, followed by host-cell remodeling that supports replication and dissemination. Once inside the host, they manipulate cellular pathways, metabolically adapt to their niche, and evade immune responses. Understanding these interactions is essential not only for defining mechanisms of pathogenesis, but also for identifying next-generation therapeutics and vaccines.

**FIGURE 1:**
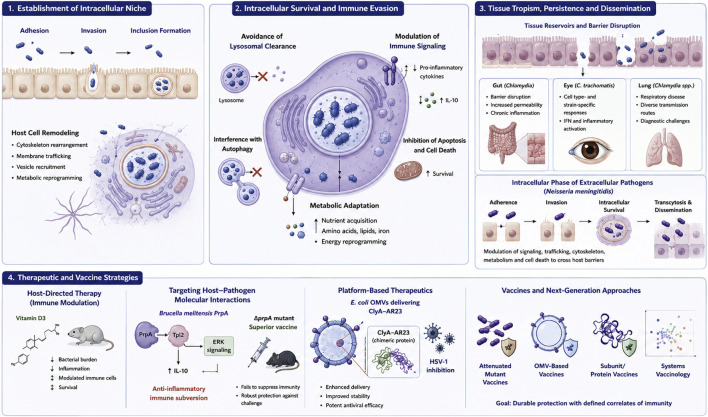
Host–pathogen interactions in intracellular bacterial infections and therapeutic interventions. This illustration shows the important phases in intracellular bacterial infection and the related intervention strategies. Panel 1 shows the formation of the intracellular niche, which is defined by pathogen adherence, invasion, immune response, host cell remodeling, cytoskeletal rearrangement, endothelium recruitment, and metabolic reprogramming. Panel 2 focuses on intracellular survival and immune evasion, which includes avoiding lysosomal degradation, modulating immune signaling, reducing pro-inflammatory cytokine release, inducing IL-10, inhibiting apoptosis, interfering with autophagy, and acquiring nutrients (Zhang et al.). Panel 3 illustrates tissue tropism, persistence, and dissemination via tissue reservoirs, barrier breakdown, chronic inflammation, and pathogen transmission throughout host tissues (Ghasemian & Holland; Resta et al.). Panel 4 highlights therapeutic and vaccination options to improve protection against intracellular infections, such as host-directed therapeutics, immune-modulating interventions, targeted delivery systems, and next-generation vaccine platforms like attenuated, recombinant, and subunit vaccines (Mensitieri et al.; Tarantino et al.; Zhang et al.). Figure was generated using Biorender.

This Research Topic, “Host-Pathogen Interactions in Intracellular Bacteria: Mechanisms, Evasion Strategies, and Therapeutic Insights,” brings together original research and review articles examining how obligate and facultative intracellular bacteria interact with host tissues, immune cells, epithelial barriers, and intracellular signaling networks. Collectively, the articles highlight diverse intracellular lifestyles, from *Chlamydia* species, an intracellular pathogen, to *Neisseria meningitidis*, an extracellular pathogen that engages intracellular phases. The collection also emphasizes systems-level approaches and host-directed therapeutic strategies that modulate immunity rather than targeting the pathogen alone.

A major focus of this Research Topic is *Chlamydia*, obligate intracellular bacteria that illustrates the complexity of long-term persistence, tissue tropism, and immune-mediated pathology. Wan et al. used single-cell RNA sequencing to investigate long-term *Chlamydia* colonization in the mouse gut, which could serve as a reservoir for persistent infection (Wan et al.). Their study revealed that long-term intestinal colonization was associated with epithelial barrier disruption, increased intestinal permeability and chronic mucosal dysfunction. These findings suggest that *Chlamydia* persistence in the gut is not merely a passive colonization state, but an active host-pathogen interaction that remodels the intestinal microenvironment and promotes bacterial persistence.

Ghasemian and Holland performed comparative transcriptomic profiling of human conjunctival epithelial cells and macrophages infected with ocular *Chlamydia trachomatis* genovars A and B, revealing striking cell type- and strain-specific transcriptional programs (Ghasemian & Holland). Conjunctival epithelial cells showed progressive transcriptional activation over time, whereas macrophages exhibited an early peak followed by attenuation. Notably, genovar B induced stronger interferon-stimulated gene and inflammatory pathway activation in epithelial cells, providing molecular insight into genovar-specific differences in trachoma severity and ocular tissue damage.

Tu et al. broadened the discussion of *Chlamydia* beyond classical urogenital and ocular disease by reviewing the contribution of *Chlamydia* species to community-acquired pneumonia (Tu et al.). Their review systematically evaluates evidence on *Chlamydia pneumoniae*, *Chlamydia psittaci*, and *C. trachomatis* as respiratory pathogens with distinct transmission routes, host tropisms, and clinical manifestations. The review underscores the diagnostic challenges posed by non-specific clinical presentations and highlights the need for improved molecular diagnostics, stratified treatment strategies, and broader recognition of *Chlamydia* as a contributor to respiratory disease.

Resta et al. provided a comprehensive review of *Neisseria meningitidis*, a pathogen traditionally viewed as extracellular but capable of an intracellular phase, summarizing how meningococci interact with epithelial and endothelial barriers (Resta et al.). The authors discuss mechanisms of transcellular and paracellular crossing, intracellular survival, vacuolar or cytoplasmic localization, and bacterial modulation of signal transduction, membrane trafficking, cytoskeletal dynamics, metabolism, and programmed cell death. This review highlights how intracellular phases may contribute to meningococcal persistence, barrier traversal, and invasive disease.

In addition to defining mechanisms of pathogenesis, this collection addresses host-directed and platform-based therapeutic strategies. Tarantino et al. investigated vitamin D3 in control of *Salmonella Typhimurium* infection in an *in vivo* mouse model (Tarantino et al.). As *Salmonella* survives within host immune cells and promotes inflammatory tissue damage, the authors targeted host immunity and demonstrated that active vitamin D3 reduced bacterial burden, attenuated histopathological inflammation, modulated immune cell profiles, and prolonged survival. These findings support host immune modulation as a strategy to improve control of intracellular bacterial infections while limiting immunopathology.

Zhang et al. dissected the molecular strategy of *Brucella melitensis*, demonstrating that the bacterial protein PrpA interacts with the host kinase Tpl2, hijacking the ERK signaling pathway to regulate anti-inflammatory cytokine IL-10 (Zhang et al.). This represents active immune subversion, allowing *Brucella* to create conditions favorable for chronic infection. Critically, this discovery is immediately translatable. A *prpA* mutant not only fails to suppress immunity but also acts as a superior vaccine, offering robust protection against virulent challenge.

For the first time, Mensitieri et al. has shown *Escherichia coli* derived outer membrane vesicles (OMVs) can be utilised as antiviral presentation platforms by combining expression of cytolysin A (Cly A) chimeric protein with antiviral peptide, AR23 (Mensitieri et al.). When tested against HSV-1, ClyA-AR23 OMVs effectively disrupted viral replication in a dose-dependent manner. This study establishes a promising strategy to overcome key limitations of antimicrobial peptides, including poor stability, limited bioavailability, and suboptimal therapeutic efficacy, by engineering an efficient OMV based delivery platform that enhances their functional performance and translational potential.

Together, the articles in this Research Topic reveal several converging principles. First, intracellular bacterial pathogenesis is highly context dependent: the same pathogen may induce distinct responses depending on tissue site, host cell type, infection stage, and strain background. Second, persistence often depends on active remodeling of host cell architecture and immune communication, including epithelial barrier disruption, altered vesicular trafficking, modulation of apoptosis, and evasion of lysosomal or inflammatory clearance pathways. Third, modern omics technologies dissect these interactions at greater resolution, identifying cell-specific signatures that bulk approaches often miss. Finally, therapeutic development must move beyond pathogen killing alone to include host-directed interventions, immune recalibration, targeted delivery systems, and vaccines designed around mechanistic correlates of protection.

Despite significant progress, many important questions remain unresolved. The molecular determinants that allow bacteria to shift between acute infection, persistence, latency, and dissemination are still incompletely understood. For pathogens such as *Chlamydia*, the relationship between tissue reservoirs, reinfection, chronic inflammation, and long-term sequelae requires further investigation. For *N. meningitidis*, the contribution of intracellular phases to invasive disease remains an important area for future study. Moreover, host-directed therapies must be carefully optimized to enhance protective immunity without exacerbating inflammatory pathology.

In summary, by integrating mechanistic microbiology, host-cell interaction biology, transcriptomics, barrier biology, and therapeutic innovation, this Research Topic highlights the dynamic nature of host-pathogen interactions and provides a timely perspective on how intracellular bacteria persist, evade host defenses, and cause disease.

